# Two Major Clades of Bradyrhizobia Dominate Symbiotic Interactions with Pigeonpea in Fields of Côte d'Ivoire

**DOI:** 10.3389/fmicb.2016.01793

**Published:** 2016-11-11

**Authors:** Romain K. Fossou, Dominik Ziegler, Adolphe Zézé, François Barja, Xavier Perret

**Affiliations:** ^1^Microbiology Unit, Department of Botany and Plant Biology, University of GenevaGeneva, Switzerland; ^2^Mabritec AGRiehen, Switzerland; ^3^Laboratoire de Biotechnologies Végétale et Microbienne, Unité Mixte de Recherche et d'Innovation en Sciences Agronomiques et Génie Rural, Institut National Polytechnique Félix Houphouët-Boigny (INPHB)Yamoussoukro, Côte d'Ivoire

**Keywords:** nitrogen fixation, *Cajanus cajan*, nodulation, bio-inoculant, smallholders, MALDI-TOF MS

## Abstract

In smallholder farms of Côte d'Ivoire, particularly in the northeast of the country, *Cajanus cajan* (pigeonpea) has become an important crop because of its multiple beneficial facets. Pigeonpea seeds provide food to make ends meet, are sold on local markets, and aerial parts serve as forage for animals. Since it fixes atmospheric nitrogen in symbiosis with soil bacteria collectively known as rhizobia, *C. cajan* also improves soil fertility and reduces fallow time. Yet, seed yields remain low mostly because farmers cannot afford chemical fertilizers. To identify local rhizobial strains susceptible to be used as bio-inoculants to foster pigeonpea growth, root nodules were collected in six fields of three geographically distant regions of Côte d'Ivoire. Nodule bacteria were isolated and characterized using various molecular techniques including matrix-assisted laser desorption/ionization time of flight (MALDI-TOF) mass spectrometry (MS) and DNA sequencing. These molecular analyses showed that 63 out of 85 nodule isolates belonged to two major clades of bradyrhizobia, one of which is known as the *Bradyrhizobium elkanii* super clade. Phylogenies of housekeeping (16S-ITS-23S, *rpoB*) and symbiotic (*nifH*) genes were not always congruent suggesting that lateral transfer of nitrogen fixation genes also contributed to define the genome of these bradyrhizobial isolates. Interestingly, no field-, plant-, or cultivar-specific effect was found to shape the profiles of symbiotic strains. In addition, nodule isolates CI-1B, CI-36E, and CI-41A that belong to distinct species, showed similar symbiotic efficiencies suggesting that any of these strains might serve as a proficient inoculant for *C. cajan*.

## Introduction

Unlike cereals that must rely almost exclusively on chemical fertilizers to sustain their growth, legume crops can take advantage of nitrogen-fixing associations with soil bacteria that are collectively known as rhizobia. During these beneficial interactions, rhizobia exchange reduced forms of atmospheric nitrogen provided to host plants against all the macro- and micro-nutriments required to sustain symbiotic nitrogen fixation (Udvardi and Poole, 2013). Unlike free-living diazotrophs, many rhizobia must first establish persistent intracellular colonies inside plant cells of root nodules prior to the activation of functions required for nitrogen fixation (Masson-Boivin et al., 2009). During the nodulation process that allows rhizobia to infect nodule cells, host plants actively screen infecting bacteria for pathogens, or non-symbiotic strains via the exchange of multiple molecular signals (Perret et al., 2000; Oldroyd et al., 2011; Nelson and Sadowsky, 2015). In spite of such selectivity in the pairing of plant-bacteria symbionts, rhizobia belong to remarkably diverse microbial genera, possibly because lateral transfer of symbiotic genes contributes to diversify the number of rhizobial solutions to the legume needs (Masson-Boivin et al., 2009). In addition to conjugative symbiotic plasmids as in *Sinorhizobium* (*Ensifer*) *fredii* strain NGR234 (Freiberg et al., 1997), lateral transfer of genomic islands was shown to promote dispersal of symbiotic genes to various non-symbiotic recipient soil bacteria including mesorhizobia (Ramsay et al., 2006; Nandasena et al., 2007) and bradyrhizobia (Barcellos et al., 2007; Okubo et al., 2012). Such diversity of potential micro-symbionts in soils often reduces the beneficial effects of bio-inoculants on legume crops (Denton et al., 2002; Fening and Danso, 2002; Nandasena et al., 2007).

Amongst the many legumes that are cultivated worldwide, *Cajanus cajan* L. is primarily grown as a food crop by smallholder farmers of many tropical and subtropical regions (Varshney et al., 2010). Often cultivated as a sole crop or in mixed cropping systems with short-maturing cereals or legumes, *C. cajan* is valued for its protein-rich seeds that are used for human consumption and for its aerial parts that find application as forage and fodder (Varshney et al., 2010). In 2013, the annual production of *C. cajan* was estimated at 4.9 × 10^6^ tons worldwide, of which >60% was grown in India alone (FAO statistics; http://faostat3.fao.org/browse/Q/QC/E). Being consumed as green peas or dry grains, *C. cajan* has become the main source of proteins for more than a billion people worldwide, and a cash crop that supports millions of resource-poor farmers in various developing countries (Mula and Saxena, 2010; Varshney et al., 2012). Traditionally grown as food to make ends meet in the northeast of Côte d'Ivoire, *C. cajan* is also cultivated for human consumption and animal feed in other parts of the country with distinct pedoclimatic characteristics (Ndabalishe, 1995). For example, in the political capital Yamoussoukro, pigeonpea seeds can be purchased on local markets and used as chicken feed (Fossou et al., 2012) while in the northern parts of the country late maturing varieties are favored as forage during the dry season (Charpentier et al., 1999). In addition, *C. cajan* was also tested as a plant to improve soil fertility in savannahs (Koné et al., 2012), to reduce the erosion of agricultural soils with 2–6% slopes (Charpentier et al., 1999) and as an intercrop in upland rice cropping systems (Akanvou et al., 2002). In the context of the Heifer International Project, the ILRI 16555 cultivar of the International Livestock Research Institute (ILRI) in Ethiopia was introduced in 2004 in the center of Côte d'Ivoire as a perennial and vegetative legume for providing forage to cattle in cotton-based systems (Poussy Sébé, personnal communication). The ability of *C. cajan* to fertilize soils also justifies its use in smallholder farms of Asia and Africa where chemical fertilizers often are unaffordable. In such fields, *C. cajan* was measured to derive 65% of its nitrogen from biological nitrogen fixation (BNF) (Herridge et al., 2008) and to release into soils a calculated 30–40 kg/ha of nitrogen residues (Sheldrake and Narayanan, 1979).

Studies aiming at characterizing microbial symbionts of pigeonpea confirmed that, regardless the country in which the study was carried out, members of slow-growing bradyrhizobia species were favored over fast-growing bacteria. For examples, *Bradyrhizobium elkanii* was found to be the dominant symbiont in fields of Trinidad-Tobago (Ramsubhag et al., 2002) while several slow-growing rhizobial strains nodulating *C. cajan* in the Dominican Republic were reported to be closely related to *Bradyrhizobium yuanmingense* CCBAU 11071^T^ (Araujo et al., 2015). Pigeonpea was also found to form efficient nodules with a number of fast-growing isolates including members of the *Rhizobium* (Wolde-Meskel et al., 2005; Degefu et al., 2013) or the *Ensifer* (formerly *Sinorhizobium*) genera (Stepkowski et al., 2003), amongst which strain NGR234 is well known for its exceptional broad host-range (Pueppke and Broughton, 1999). Yet, in spite of its importance in rural economies, little was known on root nodule bacteria (RNB) that form beneficial symbioses with pigeonpea in Côte d'Ivoire.

This work thus aimed at (i) establishing a catalog of rhizobia isolated from nodules of pigeonpea (*Cajanus cajan* L.) collected in Ivorian fields and at (ii) comparing the symbiotic efficiencies of representative nodule isolates. Until recently, reliable identification and characterisation of rhizobial isolates relied mostly on comparative analysis of marker genes. Often used as the primary phylogenetic marker, the 16S ribosomal RNA (rRNA) gene was shown to provide robust bacterial phylogenies (Schleifer, 2009) but also to lack resolving power at and below species level (Willems, 2006) as well as to result in ambiguous assignments when bacteria harbored multiple and divergent copies of 16S rDNA sequences (van Berkum et al., 2003). Multilocus sequence analysis (MLSA) of conserved protein-coding core genes was shown to overcome some of these limitations, and was successfully used to delineate species within rhizobial genera such as *Ensifer* (Martens et al., 2008), and *Bradyrhizobium* (Rivas et al., 2009) or to support the creation of the *Neorhizobium* genus (Mousavi et al., 2014). However, recent genome analyses confirmed that horizontal gene transfer and intergenic recombination often impaired taxonomic classification (Tian et al., 2012; Zhang et al., 2012). Given its potential to facilitate and expedite the exploration of rhizobial diversity in agricultural and natural ecosystems (Ziegler et al., 2012), matrix-assisted laser desorption/ionization time of flight (MALDI-TOF) mass spectrometry (MS) has become a rapid and reliable alternative to DNA sequencing for the identification of nodule isolates (Ferreira et al., 2011; Sánchez-Juanes et al., 2013) or their classification (Jia et al., 2015). In fact, MALDI-TOF MS was found to be so efficient in discriminating between closely related strains, that a large reference database covering all of the major rhizobial genera was established (Ziegler et al., 2015).

Here we report on the sampling of pigeonpea nodules in six fields of three distant regions of Côte d'Ivoire, and the subsequent isolation and molecular characterization of nodule bacteria. Once sorted into separate phyletic clusters based upon their MALDI-TOF MS signatures, a subset of strains representing the diversity of the 85 nodule isolates was characterized at the molecular level using MLSA of 16S rDNA, internal transcribed spacer (ITS), and *rpoB* (β-subunit of RNA polymerase) chromosomal marker genes. Symbiotic properties of representative nodule isolates were assessed by inoculating each strain separately onto a set of legume species that were grown in standard laboratory conditions, as well as by sequencing the respective *nifH* genes often used to study the evolution of nitrogen-fixing bacteria (Raymond et al., 2004).

## Materials and methods

### Bacterial growth conditions

Strains that were isolated during this study are listed in Table S1. Nodule isolates and the spontaneous rifampicin-resistant derivative of reference *Sinorhizobium* (*Ensifer*) *fredii* strain NGR234 (Stanley et al., 1988) were grown at 27°C in/on tryptone-yeast (TY) (Beringer, 1974) or rhizobial minimal medium supplemented with 12 mM succinate (RMS) as sole carbon source (Broughton et al., 1986).

### Isolation of nodule bacteria

Fields and number of plants sampled in each of them are listed in Table 1. Once collected from roots, nodules were desiccated in plastic tubes containing silica gel and stored at 4°C until further analysis. Prior to isolation of nodule bacteria, plant tissues were incubated overnight at 4°C in sterile double distilled water (ddH_2_O). Once nodules were rehydrated, the remaining soil traces were carefully removed. Sterilization of nodule surface was carried out using one initial 3 min incubation in 70% (v/v) EtOH followed by 3 min in 4% (w/v) sodium hypochlorite solution and thorough washing with sterile ddH_2_O. Once sterilized, nodules were rolled onto TY agar plates (TYA) to check for remaining surface contaminants. Each nodule was then crushed into 50 μl of sterile ddH_2_O, and an aliquot of the nodule lysate was used to inoculate a Petri dish containing TYA. All plates were incubated at 27°C and bacterial growth monitored each day. When nodule isolates started growing, a sample was used to inoculate a liquid culture which, following subsequent serial dilutions was used to isolate single colonies on RMS. Thus, all of the isolates described in this study were purified from a single colony.

**Table 1:** **Geographic positions and main characteristics of *C. cajan* sampled fields**.

**Locality**	**Field**	**Position**	**Vegetation cover**	**Sampled**	
				**Plants**	**Nodules**
Kossou-Bouafla	1	N 7°17′45″ W 5°49′00″	unplowed 8 years-old fallow with sparse *C. cajan* trees.	5	26
	2	N 7°18′23″ W 5°49′06″	unplowed 8 years old fallow with dense *C. cajan* cover.	5	26
Yamoussoukro	3	N 6°51′19″ W 5°14′38″	sparse 3–4 years old *C. cajan* plants in unkept backyard.	3	11
	4	N 6°51′02″ W 5°13′31″	3 years-old plowed field of *C. cajan* intercropped with *Jatropha curcas*.	4	21
Bondoukou	5	N 7°55′28″ W 2°57′45″	1 year-old plowed field of *C. cajan* intercropped with *Dioscorea* sp.	6	48
	6	N 7°56′50″ W 2°56′26″	1 year-old unplowed field inter-cropped with *Manihot esculenta*.	5	39

### Molecular characterization of nodule isolates

To obtain a preliminary identification and sorting of nodule isolates, each strain was cultivated separately on TYA and at 27°C. When growth was sufficient, each isolate was analyzed in quadruplicate by mass spectrometry as described in Ziegler et al. (2015). Briefly, free-living bacteria were spotted onto MALDI steel target plates, overlaid with 1 μl of 25% formic acid, air-dried, and again overlaid with 1 μl of matrix solution consisting of saturated alpha-cyano-4 hydroxycinnamic acid (CHCA; Sigma-Aldrich, Buchs, Switzerland) in 33% acetonitrile (Sigma-Aldrich), 33% ethanol and 3% trifluoroacetic acid (TFA). Once dried, bacterial spots were analyzed with MALDI-TOF Mass Spectrometer Axima™ Confidence machine (Shimadzu-Bio- tech, Kyoto, Japan) using the linear positive detection mode, a laser frequency of 50 Hz and a mass range of 3–15 kDa. For each isolate, spectra consisting of 50–100 protein masses were averaged and processed using the Launch-pad™ 2.8 software (Shimadzu-Biotech). For identification purposes, mass spectra were matched against the PARPM and the “rhizobia-specific module” of the SARAMIS™ databases that were described previously (Ziegler et al., 2015). When isolates did not match any of the reference rhizobial strains, the search was extended to bacteria included in a proprietary and enlarged version of the SARAMIS™ database (Mabritec AG). For hierarchical clustering of protein masses, a binary matrix listing presence/absence of masses was generated using the SARAMIS Superspectra tool (see Table S5). Results were then imported into the PAleontological STatistics (PAST) software (Natural History Museum, Oslo University, Norway). Multivariate neighbor-joining cluster analyses with similarity-distances were calculated using correlation algorithm in PAST software (Feltens et al., 2010). Cluster analysis was saved as a Nexus file and imported into FigTree software to generate a circular tree. For sequencing analyses, genomic DNA (gDNA) of each of the selected isolates was prepared as in Chen and Kuo (1993). Amplifications of the 16S rDNA, ITS, *nifH*, and *rpoB* sequences were carried out on a T-Gradient thermocycler (Biometra, Göttingen, Germany) in 50 μl PCR reactions containing 50 ng of gDNA, 0.2 mM dNTPs, 1 unit Taq polymerase and 1 μM final concentration of each of the corresponding primers listed in Table S2. Prior to sequencing, amplified products were separated onto agarose gels and purified using the NucleoSpin gel and PCR clean-up kit (Macherey-Nagel AG, Oensingen, Switzerland). Purified amplicons were sequenced at Microsynth AG (Balgach, Switzerland) using Sanger sequencing. High quality sequences were assembled into double stranded DNA sequences that were manually curated for ambiguous nucleotide positions and primer sequences. Except for strains CI-16A, CI-34D1, and CI-34F for which only 16S rDNA sequences were obtained, the corresponding 16S and ITS sequences of selected nodule isolates were deposited in GenBank as assembled 16S-ITS-partial 23S sequences. All DNA sequences used in this study were deposited in GenBank under the accession numbers that are listed in Table S2.

### Phylogenetic analyses

Putative phylogenetic relationships were inferred with the MEGA software version 6 (Tamura et al., 2013) using DNA sequences of selected isolates listed in Table S2. The corresponding 16S-ITS-23S, *nifH*, and *rpoB* sequences of the reference strains *Bradyrhizobium* genosp. CB756, *B. diaozoefficiens* USDA 110^T^, *B. elkanii* USDA 76^T^ and USDA 3259, *B. japonicum* USDA 6^T^, and *B. pachyrhizi* BR3262 were retrieved from GenBank. Reference strains were selected on the basis of taxonomic proximity to pigeonpea isolates and for best anchoring of tree branches. DNA sequences were aligned with ClustalW (for *nifH* and *rpoB*) or MUSCLE (for rRNA operon sequences) as implemented in MEGA, and manually corrected when needed. Phylogenetic trees were inferred using the neighbor-joining (NJ) method with statistical support calculated from 1000 bootstrap replicates. The best-fit nucleotide substitution model was selected according to the Bayesian information criterion (Schwarz, 1978): Tamura-Nei + Gamma (TN93+G) parameters for *rpoB* and 16S-ITS-23S sequences and Tamura 3 + Gamma (T92+G) parameters for *nifH*. In all trees, a corresponding sequence of *S. fredii* strain NGR234 was used as outgroup.

### Plant assays

Seeds of *C. cajan* cultivars ILRI 16555 and “Light Brown” were surface-sterilized using concentrated sulphuric acid for 10 min, 0.1% (v/v) Tween 20 for 5 min and 5% hydrogen peroxide for 5 min, with intermediate washing steps using sterile ddH_2_O. Seeds of *Leucaena leucocephala, Macroptilium atropurpureum* cv. Siratro, *Tephrosia vogelii, Vigna radiata* cv. King, and *Vigna unguiculata* cv. Red Caloona were surface sterilized as described previously (Fumeaux et al., 2011). To germinate, surface-sterilized seeds were incubated for two to three days in the dark, at 27°C and on B&D agar plates. Once germinated, seedlings were planted in Magenta jars (two plants per jar) containing vermiculite (Lewin et al., 1990), and watered using nitrogen-free B&D solution (Broughton and Dilworth, 1971). When plantlets emerged from the vermiculite, each of them was inoculated with 200 μl of a water solution containing 2 × 10^8^ freshly grown bacteria. Plants were grown in controlled conditions with a light phase of 12 h, a day temperature of 27°C, a night temperature of 20°C and 60–70% humidity. Except when stated otherwise, plants were harvested at 42 days post inoculation (dpi). Symbiotic proficiency of each inoculant was assessed using as parameters the nodule number, total nodule fresh weight, dry weight of shoots, and presence/absence of leghemoglobin inside nodules or pseudonodules. The promiscuous *S. fredii* strain NGR234 was used as a control inoculum since it was shown to nodulate and fix nitrogen with all plants tested in this study (Perret et al., 1999; Pueppke and Broughton, 1999). Identity of each inoculant was confirmed by PCR amplification of ITS sequences using ITS-For2 and ITS-Rev2 primers and aliquots of nodule lysates as template.

### Scanning electron microscopy

To minimize changes in cell structures, bacteria were fixed directly on an agar plate, for 1 h at room temperature using a final concentration of glutaraldehyde of 2% (v/v). After three successive washes with 1 ml of 66 mM Sörensen's sodium phosphate buffer (KH_2_/Na_2_HPO_4_2H_2_O, pH 6.8), cells were post-fixed in 2% (w/v) osmium tetroxide solution for 1 h. After another wash with Sörensen's buffer, samples were dehydrated by successive immersions of 10 min in solutions of respectively 25, 50, 75, and 95% (v/v) ethanol at 4°C. Samples were then washed three times for 10 min in 100% ethanol at room temperature, and finally incubated in isoamyl acetate for 1 h. Dehydrated samples were then mounted on aluminum mounts with silicon wafers, coated with gold and observed with a JEOL JSM 7001FA field emission scanning microscope.

### Basic soil properties

Physical and chemical properties of soil samples were determined by a commercial soil analysis laboratory (Soil-Conseil, Gland, Switzerland) using standard methods (ISO 17025). Briefly, samples were homogenized, dried for 48 h at 40°C and sieved with fraction <2 mm being used for subsequent analyses. Soil texture was determined by sedimentation once the humus was destroyed by H_2_O_2_. Soil type was determined using the standard USDA particle-size classification. Soluble elements (Ca, Cu, Fe, K, Mg, Mn, P, and Zn) were extracted with water and composition determined by spectrophotometry and spectrometry. pH was measured using 20 g of 2 mm sieved soil samples stirred in 50 ml ultrapure H_2_O. Organic matter (%) was determined by oxidizing soil carbon with a mixture of K_2_Cr_2_O_7_ 1N and H_2_SO_4_ 95–98%, total nitrogen (%) was measured using the Kjeldhal method, and available nitrogen was extracted with 0.01 M CaCl_2_ solution and measured by spectrophotometry.

## Results

### Characteristics of sampled fields

**Figure 1:**
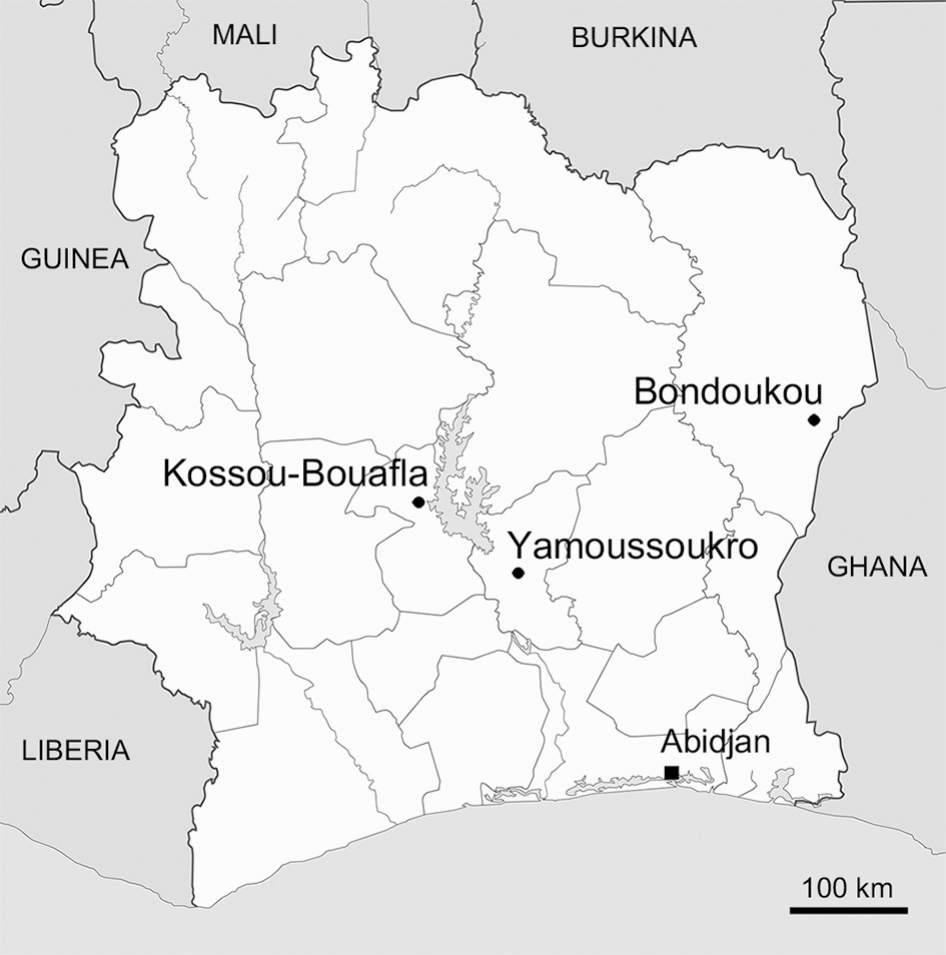
**Regional map of Côte d'Ivoire with positions of the three sampled sites**. Geographic coordinates in degrees, minutes, and seconds of the sampled fields in Kossou-Bouafla, Yamoussoukro, and Bondoukou are given in Table 1.

Two separate fields were sampled in each of the three localities that were selected for their different records of *C. cajan* cultivation (see Figure 1). Close to the northeast city of Bondoukou, next to the border with Ghana, cultivation of pigeonpea has been established for many years with inhabitants having given vernacular names in local dialects to this crop (e.g., “*Kapkô*” in Koulango). The two sampled fields of Bondoukou covered an area of 0.5 and 2 ha, and were intercropped with yam (*Dioscorea* sp.) and cassava (*Manihot esculenta)*, respectively. In this area of Côte d'Ivoire, seeds of pigeonpea found on local markets had diverse sizes and colors (ranging from whitish to brown), indicating that farmers favored growth of different landraces rather than a specific cultivar. This contrasted with the *C. cajan* plants grown in the two fields of Kossou-Bouafla and that were exclusively offsprings of the ILRI 16555 cultivar. ILRI 16555 was introduced in 2004 via a program sponsored by the Heifer International charity for fostering the use of perennial cultures of pigeonpea as forage to cattles in cotton-based agricultural systems. Accordingly, seeds held by farmers in Kossou-Bouafla were uniformly brown. The remaining two fields of 0.5 and 1.5 ha, were sampled in the outskirts of the capital Yamoussoukro. They were used for planting uncharacterized cultivars of *C. cajan*. The field covering 1.5 ha was intercropped with *Jatropha curcas* plants, and was used for producing pigeonpea seeds for poultry feeding. In each of the six sampled fields and within 1.5 m from the trunk, root systems of three to six plants were cleared of soil. Only nodules attached to the cleared root system were collected. Once cleared of most soil particles, nodules that belonged to the same root system were stored together into a single desiccating vial and kept at 4°C until further processing. In total, 171 root nodules belonging to 28 plants were collected in the six designated fields.

### Isolation and MALDI-TOF MS characterisation of nodule bacteria

**Figure 2:**
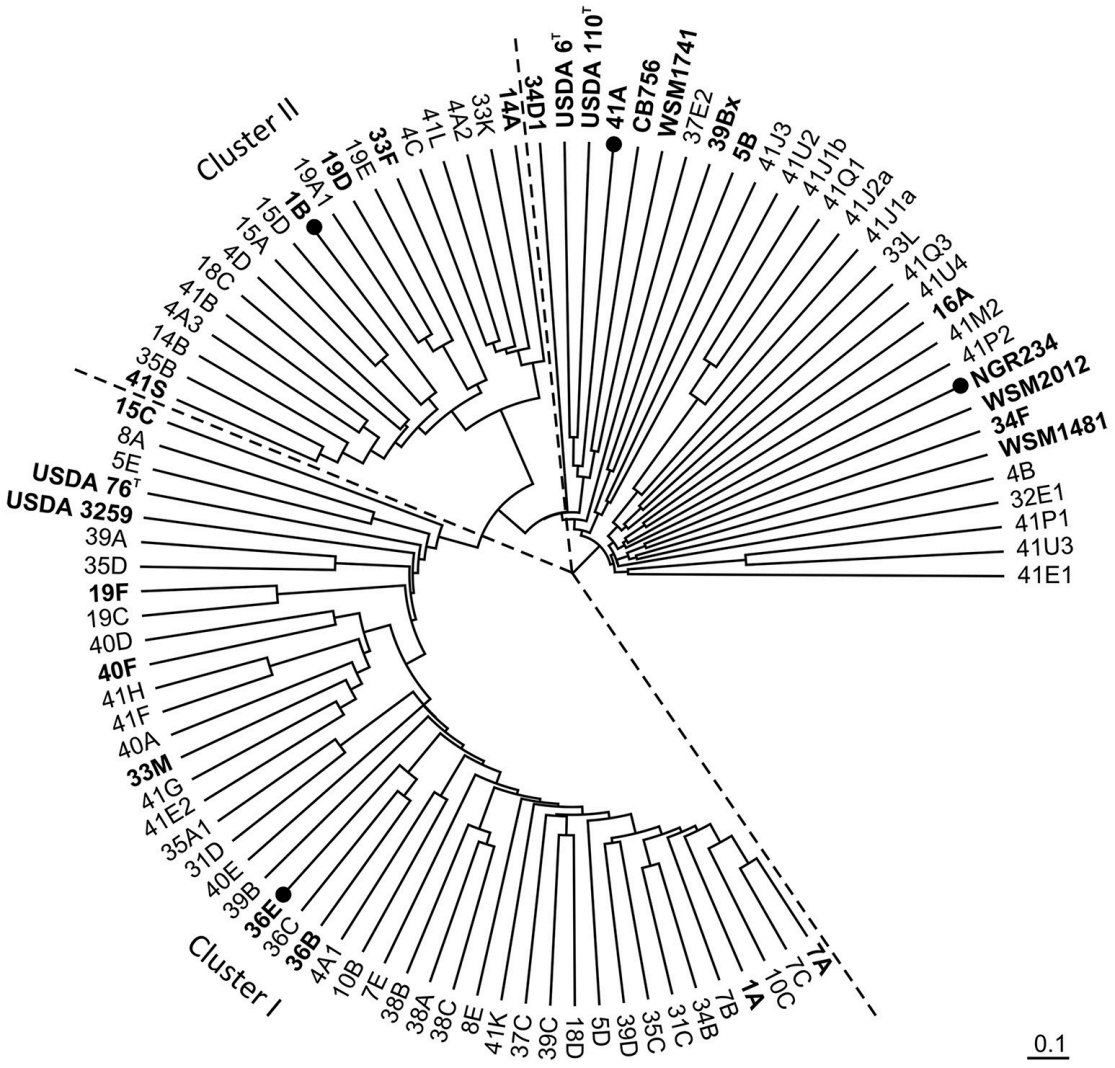
**Unsupervised hierarchical cluster analysis of MALDI-TOF MS signatures for nodules isolates and selected reference strains using the characteristic protein masses comprised between *m/z* 3 and 15 kDa that are listed in Table S5**. Nodule isolates from Côte d'Ivoire (CI−) are only referred to by the strain number. Reference strains *Bradyrhizobium* sp. WSM1741, *Bradyrhizobium* genosp. CB756, *B. diaozoefficiens* USDA 110^T^, *B. elkanii* USDA 76^T^ and USDA 3259, *B. japonicum* USDA 6^T^, *Rhizobium leguminosarum* bv. *viciae* WSM1481, *R. leguminosarum* bv. *trifolii* WSM2012, *S. fredii* NGR234 as well as nodule isolates for which DNA sequences were obtained are marked in bold. Strains for which symbiotic proficiency on *C. cajan* was reported in Table 2 are marked with a black circle. Dashed lines delimitate the two major clusters of nodule isolates.

A total of 85 nodule isolates were recovered and purified as single colonies. Initial identification was performed with MALDI-TOF MS as in Ziegler et al. (2015). Mass spectral analyses did not only provide a preliminary identity at the genus or species level for most of the nodule isolates, but also allowed for a cluster analysis of MS signatures. As shown in Figure 2, 62 isolates (73%) clustered into two clearly separated groups of strains (clusters I and II). The larger of these two groups included the reference *B. elkanii* strains USDA 76^T^ and USDA 3259 as well as 43 isolates of *C. cajan* nodules (lower left quadrant in Figure 2). The second cluster consisted of 19 closely related isolates (Figure 2, upper left quadrant) identified by MALDI-TOF MS as *bradyrhizobia* sp. The remaining 23 isolates were extremely diverse (Figure 2, upper-right corner), with only three strains (CI-5B, -39Bx, and CI-41A) sharing rhizobia-like features (Table S1).

### 16S rRNA gene sequencing to confirm the identities of nodule isolates

To verify that the initial identification and clustering of nodule bacteria was accurate, several isolates were selected on the basis of (i) their respective position in the cluster tree shown in Figure 2, (ii) the field/soil in which nodules were found, and (iii) the bacterial identification (or absence of it) obtained via MALDI-TOF MS. Accordingly, a subset of 8 strains (CI-1A, -7A, -15C, -19F, -33M, -36B, -36E, and CI-40F) was selected amongst the 43 isolates initially identified as *B. elkanii* (cluster I). CI-1B, -14A, -19D, -33F, and CI-41S were chosen to represent the 19 bradyrhizobia that formed the second cluster of related strains, and six (CI-5B, -16A, -34D1, -34F, -39Bx, and CI-41A) of the 23 unrelated strains were retained for 16S rDNA sequencing. Except for strains CI-5B and CI-39Bx, of which the 16S genes matched those of *Rhizobium leguminosarum* bv. *viciae* and *Rhizobium* sp. strain JGI 0001005-K05 respectively, 16S rDNA sequencing confirmed the identifications obtained with mass spectra analyses (see Table S1). For example, the 8 isolates selected from cluster I were found to carry 16S rRNA genes identical to those of several members of the *B. elkanii* super clade (Aserse et al., 2012) including *B. elkanii* type strain USDA 76^T^, *B. pachyrizi* strain PAC48^T^ and *Bradyrhizobium tropiciagri* CNPSo 1112^T^. Isolates CI-1B, -14A, -19D, -33F, and CI-41S of cluster II shared identical 16S ribosomal sequences that were 99.7% similar to the same type strains USDA 76^T^, PAC48^T^ and CNPSo 1112^T^, while the extremely slow-growing isolate CI-41A was found to be closely related to *B. liaoningense*. As to isolates CI-16A, -34D1, and CI-34F that were not identified via MALDI-TOF mass spectrometry, the respective 16S rRNA sequences best matched those of *Paenibacillus sp*. JDR-2 (accession number CP001656.1), *Brevibacillus reuszeri* DSM 9887 (LGIQ01000014.1) and *Bacillus soli* strain NBRC 102451 (BCVI01000121.1). These analyses further confirmed that, for those strains for which a related reference existed in the spectral databases, MALDI-TOF MS provided an accurate identification (Ziegler et al., 2015).

### Assessing diversity of *C. cajan* isolates with ITS and *nifH* sequences

Although selected isolates from the same cluster shared identical 16S rDNA sequences, topology of the cladogram shown in Figure 2 suggested genetic diversity existed within each of these clusters. To examine whether selected isolates from clusters I (8 out of 43 strains) and II (5 out of 19) differed genetically, the internal transcribed spacer (ITS) separating 16S and 23S rRNA genes as well as the *nifH* gene that codes for the structural Fe protein of nitrogenase were amplified and sequenced. Strains CI-5B, -39Bx, and CI-41A were also included in these analyses because all three isolates possessed rhizobia-like features (see Table S1). For many years, the ITS region has been used in population genetics and molecular systematics (Gürtler and Stanisich, 1996), in particular for taxa for which 16S rRNA gene sequences lacked resolution for closely related isolates [e.g., sphingomonads (Tokajian et al., 2016)]. NifH is an essential component of the nitrogenase complex, and its gene has been widely used to survey diazotrophs in diverse ecosystems (Zehr et al., 2003).

**Figure 3:**
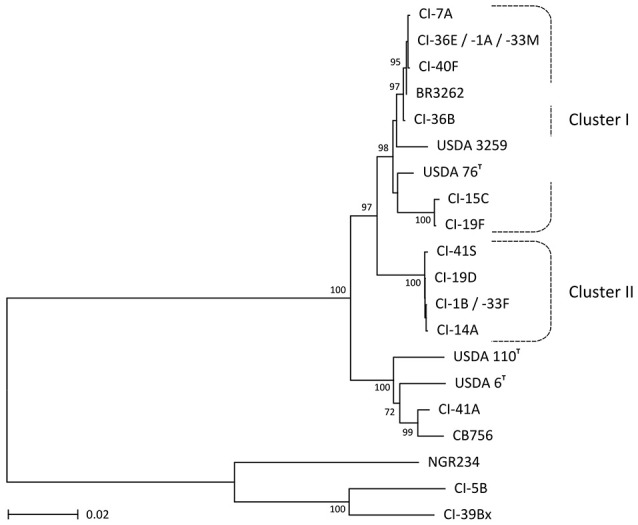
**Phylogenetic tree inferred from rRNA operon sequences corresponding to nearly complete 16S rRNA, full ITS and 5′-end of 23S rRNA sequences for a selected subset of 16 *C. cajan* nodule isolates (marked as CI- strains) and 7 reference rhizobia**. Phylogenetic tree was obtained with the NJ method, with bootstrap values issued from 1000 repetitions and only shown for those ≥70. Dashed lines mark the boundaries of the strain clusters I and II identified via MALDI-TOF mass-spectra analyses.

Once deleted of the flanking 16S and 23S rRNA sequences, the amplified ITS regions of the 16 selected nodule isolates ranged in size from 760 bp (CI-41A) to 1054 bp (CI-5B) (Table S2). Comparing these amplified ITS sequences confirmed that isolates of the *B. elkanii* cluster I were not genetically identical: CI-15C and CI-19F shared similar ITS sequences (4 mismatches over 910 nucleotides) that were longer and differed significantly from those of the remaining six isolates (801 and 802 bp long). ITS's of CI-1A, -33M, and CI-36E were identical and formed a consensus sequence, from which the ITS's of CI-7A and CI-40F differed by only one nucleotide while that of CI-36B diverged by seven bases. By contrast, and except for the one nucleotide-shorter ITS of CI-19D, ITS's of selected isolates from cluster II were 862 bp long and differed by a maximum of only six nucleotide positions. ITS regions of CI-1B and CI-33F were identical and appeared as possibly more ancient, with that of CI-41S being the most divergent of all five selected isolates. Thus, ITS analyses confirmed that, when compared to the five isolates of cluster II that showed little diversity, strains of cluster I were more diverse and could be further subdivided into two distinct groups: a set of closely related isolates (CI-1A, -7A, -33M, -36B, -36E, and -40F) with CI-19F and CI-15C forming a more distantly related outgroup. These various subgroups of nodule isolates can be clearly observed in the unrooted-phylogeny trees of 16S-ITS and partial 23S as well as *rpoB* sequences shown in Figure 3 and Figure S1.

**Figure 4:**
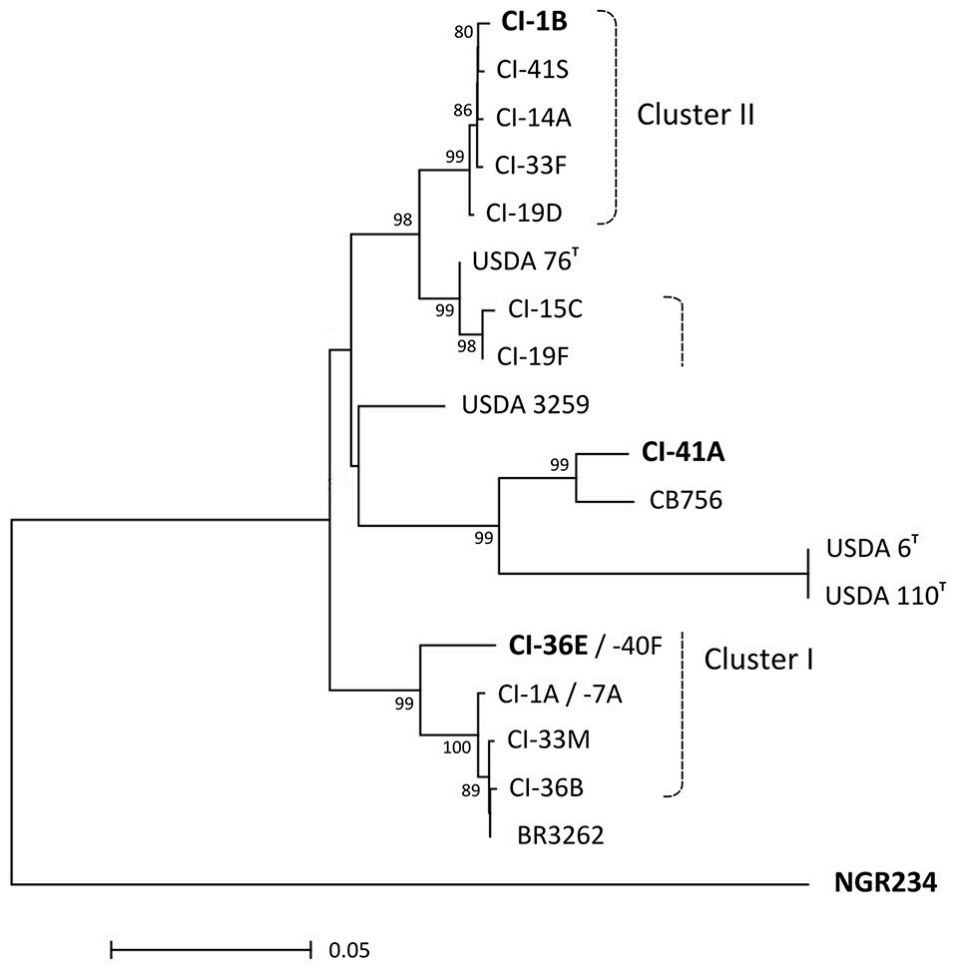
**Phylogenetic tree inferred from *nifH* genes of selected *C. cajan* nodule isolates (CI- strains) and 7 reference rhizobia**. Phylogenetic tree was obtained with the NJ method, with bootstrap values issued from 1000 repetitions and only shown for those ≥75. Dashed lines delimit the boundaries of the strain clusters I and II identified via MALDI-TOF mass-spectra analyses. The discrepant position of CI-41A and related reference CB756 strains, highlights the separation of CI-15C, and CI-19F from the remaining strains of cluster I. Strains tested for symbiotic proficiency on *C. cajan* and reported in Table 2 are shown in bold. No *nifH* amplicon was obtained for the non-symbiotic isolates CI-5B and CI-39Bx.

Diversity of selected isolates was further confirmed by comparing *nifH* sequences. With a maximum of four diverging nucleotide positions, NifH coding sequences of selected cluster II isolates (CI-1B, -14A, -19D, -33F, and CI-41S) were closely related to and did best match the *nifH* gene of *B. elkanii* type strain USDA 76 (see Figure 4). In contrast, *nifH* sequences of cluster I strains fell into two distinct subgroups, one of which included isolates CI-15C and CI-19F. Unlike what was observed in the 16S-ITS-23S phylogenetic tree (Figure 3), *nifH* of CI-15C and CI-19F were closer to corresponding sequences of cluster II isolates than to those of the remaining cluster I strains (see Figure 4). In fact, the best match for *nifH* of CI-1A, -7A, -33M, -36B, -36E, and CI-40F was the corresponding gene of *Bradyrhizobium pachyrhizi* strain BR3262. As in Figure 3, CI-41A stood out from cluster I and cluster II isolates, with a best match for *nifH* in *Bradyrhizobium* genosp. CB756 (SA-4). Thus, except for the discrepant *nifH* of CI-15C and CI-19F, the 16S-ITS-partial 23S and *nifH* phylogenies were similar.

### Testing the symbiotic properties of a subset of *C. cajan* nodule isolates

Proficiency of representative CI-1B (for cluster II), CI-36E (cluster I), CI-41A (*Bradyrhizobium* sp. isolate) and control NGR234 strains was compared using offsprings of self-crossed ILRI 16555 and “Light Brown” plants collected in Côte d'Ivoire. As shown in Table 2 and Figure S2, pigeonpea isolates were clearly more proficient symbionts than NGR234 on the selected *C. cajan* cultivars, with a shoot dry weight for plants inoculated with CI-1B, -36E, and CI-41A being at least twice as much as that of plants nodulated by NGR234. While symbiotic efficacies of CI-1B and CI-41A were found to be similar on both ILRI 16555 and “Light Brown” cultivars, CI-36E appeared slightly less efficient. Interestingly, on both ILRI 16555 and “Light Brown” cultivars NGR234 made fewer (Table 2, Figure S2) but larger nodules. For example on ILRI 16555 (test #2), NGR234 formed nodules of ca. 32 mg fresh weight each whereas plants inoculated with isolates CI-1B, -36E, and -41A carried nodules of 9.4–12.1 mg fresh weight in average. Thus, when compared to pigeonpea isolates, the lower symbiotic efficacy of NGR234 on *C. cajan* possibly resulted from a reduced ability to initiate nodule formation rather than a lower nitrogen fixation capacity once bacteria were established inside nodules.

**Table 2:** **Symbiotic properties of selected strains on two *C. cajan* cultivars grown under controlled conditions for 42 days post-inoculation**.

**Inoculum**	**Assay**	***C. cajan*** **cv. ILRI 16555**				***C. cajan*** **cv. “Light Brown”**			
		**Plants**	**mNN**	**mNFW (mg)**	**mSDW (mg)**	**Plants**	**mNN**	**mNFW (mg)**	**mSDW (mg)**
No bacteria	1	8	0.0	0.0	120.8 (±26.8)	8	0.0	0.0	74.4 (±47.9)
	2	12	0.0	0.0	123.2 (±40.6)	10	0.0	0.0	50.8 (±17.2)
CI-1B	1	12	91.7 (±22.8)	973.0 (±391.5)	2003.5 (±661.1)	11	56.5 (±17.7)^f^	1061.9 (±221.4)	1841.3 (±439.6)^h^
	2	13	119.6 (±63.0)^a^	1150.0 (±449.5)^b^	2521.8 (±803.2)^d^	14	61.5 (±20.6)	1116.7 (±324.7)^i^	2093.3 (±695.9)
CI-36E	1	12	91.3 (±46.4)	792.8 (±203.6)	2073.6 (±489.6)	12	77.6 (±26.4)^f, g^	866.8 (±265.1)	1465.6 (±373.6)^h^
	2	16	76.3 (±38.2)^a^	717.3 (±232.3)^b, c^	1814.0 (±466.8)^d, e^	16	79.9 (±32.1)	821.5 (±208.2)^i, j^	1701.4 (±508.1)
CI-41A	1	12	73.7 (±17.2)	902.2 (±410.6)	1932.4 (±741.9)	12	53.3 (±19.8)^g^	942.0 (±226.8)	1745.8 (±402.1)
	2	16	82.8 (±42.0)	999.8 (±481.3)^c^	2347.9 (±905.3)^e^	14	64.8 (±25.7)	1039.2 (±311.1)^j^	2011.4 (±621.9)
NGR234	1	12	10.1 (±4.1)	455.1 (±220.1)	959.6 (±358.1)	11	4.2 (±1.9)	219.7 (±104.9)	359.8 (±115.3)
	2	15	10.2 (±4.1)	326.5 (±134.6)	751.9 (±307.5)	13	6.8 (±4.6)	202.1 (±98.8)	328.8 (±158.1)

*The symbiotic phenotype of each inoculant is reported as the mean nodule number (mNN), mean nodule fresh weight (mNFW) and mean shoot dry weight (mSDW) per inoculated plant. With the exception of NGR234 that was found to be systematically less proficient than CI strains, pairs of values found to be statistically different at the 5% level share the same superscript letter*.

A number of additional but smaller scale nodulation assays were also carried out in order to better define the host-range and nodulation properties of pigeonpea isolates (Tables S3-A and S3-B). In addition to ILRI 16555 and “Light Brown” cultivars, the selected CI-1B, -36E, and CI-41A isolates were also found to nodulate (Nod+) and fix nitrogen (Fix+) with *Macroptilium atropurpureum* cv. Siratro, *Tephrosia vogelii, Vigna unguiculata* cv. Red Caloona (Table S3-A) and *Vigna radiata* cv. King (Table S3-B). On *Leucaena leucocephala* that forms Fix+ associations with NGR234 (Lewin et al., 1990), strains CI-1B, CI-36E, and CI-41A were found to induce only few pseudonodules that appeared to be Fix-, however. By contrast, isolates CI-5B and CI-39Bx failed to nodulate any of the hosts that were so far tested: a result consistent with the absence of successful *nifH* amplifications for both of these isolates.

## Discussion

A total of more than 170 nodules of *C. cajan* were collected in three regions and six fields of Côte d'Ivoire. In fields 1–4 of Kossou-Bouafla and Yamoussoukro, roots of pigeonpea plants carried few nodules, many of which were senescent and contained no viable rhizobia. Nevertheless, 85 isolates were recovered of which 63 were found to belong to bradyrhizobia species as shown by MALDI-TOF MS and sequencing analyses. In fact, except for the extremely slow growing isolate CI-41A related to *B. liaoningense*, the other 62 bradyrhizobia fell into two major phyletic clusters. With 43 members, cluster I was found to include at least two types of isolates found to be related to distinct *B. elkanii*-like strains: CI-1A, -7A, -33M, -36B, -36E, and CI-40F formed one subgroup while the CI-15C and CI-19F formed a second subgroup of isolates. In fact 16S rDNA-ITS-23S rDNA sequencing confirmed that the six isolates CI-1A to CI-40F were closely related to a recently sequenced strain isolated in Brazil and referred to as *B. pachyrhizi* BR3262 (Simões-Araújo et al., 2016), whereas the second subgroup made of CI-15C and CI-19F-like isolates was found to be more closely related to *B. elkanii* USDA 76^T^ (see Figure 3). Whether these two subgroups of cluster I isolates indeed belong to taxonomically distinct species remains to be determined, but nodulation assays showed that symbiotic proficiency of CI-36E and CI-15C differed notably on *V. radiata* cv. King (Table S3-B). These findings support the proposal by Aserse et al. (2012) and others (Vinuesa et al., 2008; Menna et al., 2009) that *B. elkanii* constitutes a super clade of microorganisms rather than a monophyletic species. In contrast, cluster II isolates appeared to be genetically more homogenous with for example the *nifH* sequences of bacteria being geographically as distant as CI-1B (isolated from field 1 in Kossou-Bouafla) and CI-33F (field 5 in Bondoukou) differing by only three mismatches. Thus, isolates that were found to be symbiotically proficient on pigeonpea appeared to belong to four different groups of bradyrhizobia of which CI-36E (cluster I, related to *B. pachyrhizi* BR3262), CI-15C (cluster I, related to *B. elkanii* USDA 76^T^), CI-1B (cluster II, *Bradyrhizobium* sp.) and CI-41A (extremely slow growing, *B. liaoningense*) were selected as representative strains.

By contrast, none of the remaining 22 non-bradyrhizobia isolates was found to nodulate *C. cajan*, even though CI-5B and CI-39Bx were identified as putative *Rhizobium* spp. by MALDI-TOF MS and/or 16S rDNA sequence analyses. Interestingly, scanning electron micrographs showed that CI-5B cell surface differed significantly from that of CI-1B, -36E and CI-41A (see Figure S3). Whether surface polysaccharides, which often contribute to define symbiotic properties, are responsible for such difference remains to be confirmed. *nifH* amplifications repeatedly failed for CI-5B and CI-39Bx and neither strains formed nodules on *C. cajan* cultivars or any of the legumes tested so far. Taken together these results suggest that CI-5B and CI-39Bx are possibly rhizobia on the making. Whether CI-5B and/or CI-39Bx have lost or not yet acquired genes essential for symbiosis remains to be determined, but genomic studies and mobilization of known symbiotic elements may provide conclusive answers. Whether non-bradyrhizobial nodule isolates represent contaminating soil bacteria or true nodule endophytes remains uncertain. While reports describing the isolation from root nodules of non-nodulating bacteria are becoming more frequent (Muresu et al., 2008; Deng et al., 2011; Wu et al., 2011; De Meyer and Willems, 2012), little data has documented opportunistic infections (Pandya et al., 2013; Zgadzaj et al., 2015). During isolation of bacteria from pigeonpea nodules, no evidence of surface contaminants was found following surface-sterilization of nodules. As several isolates were identified as spore-forming bacteria including *Bacillus megaterium* (CI-41J1b, -41J2a, -41Q1), *B. subtilis* (CI-41U2) and *Brevibacillus reuszeri* (CI-34D1) it cannot be excluded that spores, which are likely to resist nodule surface-sterilization procedures, germinated once nodules were squashed and rapidly overgrew endosymbiotic rhizobia. Thus, no strain growing faster than bradyrhizobia was identified amongst the current catalog of true pigeonpea symbionts from Côte d'Ivoire (Table S1).

Regardless of which field was sampled, and in spite of different records of pigeonpea cultivation for the three-targeted areas of Côte d'Ivoire, similar profiles of symbiotic strains were identified with more than two thirds of proficient nodule isolates being related to members of the *B. elkanii* super clade. No plant- or cultivar-specific effect was identified as illustrated by plant n°41 (collected in Bondoukou), which was indiscriminately nodulated by strains belonging to cluster I (5 isolates), cluster II (3) or by the single extra-slow growing isolate CI-41A. In contrast to fields of Bondoukou where pigeonpea roots carried numerous nodules, plants collected in fields of Kossou-Bouafla and Yamoussoukro had fewer nodules. To verify whether soil parameters such as pH and/or total nitrogen content were possibly responsible for the apparently low symbiotic responses observed in fields of central Côte d'Ivoire, we determined the physical and chemical properties of soil samples taken in fields 1, 2 (both in Kossou-Bouafla) and 4 (Yamoussoukro). As shown in Table S4, soil pH ranged from slightly acidic (6.3) to neutral (7.2) and thus was unlikely to block nodulation. Available nitrogen ranged from 20.4 kg/ha (field n°1) to 45.7 kg/ha (field n°2), but neither values were close to the 85 kg/ha reported for optimal growth and production of pigeonpea (van der Maesen, 2006). Levels of organic matter were mostly adequate, with nitrogen/carbon ratios <15 suggesting good rates of organic matter decomposition. While potassium levels were adequate, phosphate concentrations were found to be low but not critically so. Thus, none of the soil parameters examined reached values susceptible to block nodulation in fields of Kossou-Bouafla and Yamoussoukro. Interestingly, NPK levels were highest in field n°2 where smallholder farmers reported to have used shoot matter as a green manure. This result suggests that, in combination with improved nodulation by rhizobia, the persistent use of *C. cajan* as green manure during fallow may reduce the dependency of farmers on chemical fertilizers to boost plant productivity.

Perhaps the poor symbiotic responses observed in fields 1–4 resulted from low titers of symbiotic rhizobia in these soils. Although abundance of rhizobia was not measured in the soil samples we collected, it was remarkable that in Bondoukou where pigeonpea has been cultivated for several decades root nodules were more abundant. If a low number of proficient rhizobia was indeed the factor limiting nodulation of *C. cajan*, a bio-inoculant prepared from either one or a combination of the selected bradyrhizobial isolates could possibly improve nodulation, symbiotic nitrogen fixation and eventually increase plant productivity. In this respect, isolates CI-1B (cluster II) and CI-36E (cluster I) share a number of features expected of a local bio-inoculant: both strains (i) belong to species that are ubiquitous and thus presumably well adapted to soils of Côte d'Ivoire, (ii) are symbiotically proficient on at least two *C. cajan* cultivars, and (iii) associate and fix nitrogen with additional legume crops frequently cultivated by smallholder farmers. Whether such important characteristics effectively translate into efficient nitrogen fixation during field tests remains to be tested.

## Author contributions

Following an idea by AZ, XP and AZ wrote a research proposal. RF, AZ and XP carried out the sampling of pigeonpea nodules in fields of Côte d'Ivoire. Under the supervision of XP, RF carried out the isolation, genetic characterization and symbiotic assays of nodule bacteria. DZ analyzed nodule isolates by mass spectrometry. FB made the scanning electron micrographs. RF and XP wrote the manuscript.

### Conflict of interest statement

The authors declare that the research was conducted in the absence of any commercial or financial relationships that could be construed as a potential conflict of interest.
