# Supplementary material for: Two Major Clades of Bradyrhizobia Dominate Symbiotic Interactions with Pigeonpea in Fields of Côte d'Ivoire
Source: Front Microbiol. 2016 Nov 11;7:1793. doi: 10.3389/fmicb.2016.01793 (PMC5104742; doi:10.3389/fmicb.2016.01793)
Supplement: Supplementary file 2 [file DataSheet2.pdf]

Supplementary Material

Two major clades of bradyrhizobia dominate symbiotic interactions with pigeonpea in fields of Côte d'Ivoire

Romain K. Fossou, Dominik Ziegler, Adolphe Zézé, François Barja, Xavier Perret*

*** Correspondence:** Corresponding author: xavier.perret@unige.ch

Figure S1 - Phylogenetic tree of selected *rpoB* sequences.

Figure S2 - Symbiotic phenotypes of CI-1B, CI-36E, CI-41A and NGR234 on *C. cajan* cv. ILRI 16555.

Figure S3 - Scanning electron micrographs of whole cells of CI-1B, CI-5B, CI-36E and CI-41A isolates.

Table S1 - Identification, field of origin and known characteristics of nodule isolates.

Table S2 - List of primers used and of sequences obtained during this study.

Tables S3-A and S3-B - Symbiotic properties of selected isolates on various legumes.

Table S4 - Physical and chemical properties of soil samples collected in fields #1, 2 and 4.

Table S5 - List of masses characteristic for each of the 94 strains shown in Figure 2.

Figure S1. Phylogenetic tree of *rpoB* sequences (923 bp) for seven reference rhizobial strains and four nodule isolates (in bold) that were selected as representative of the main subgroups of *C. cajan* symbionts identified in Côte d'Ivoire.

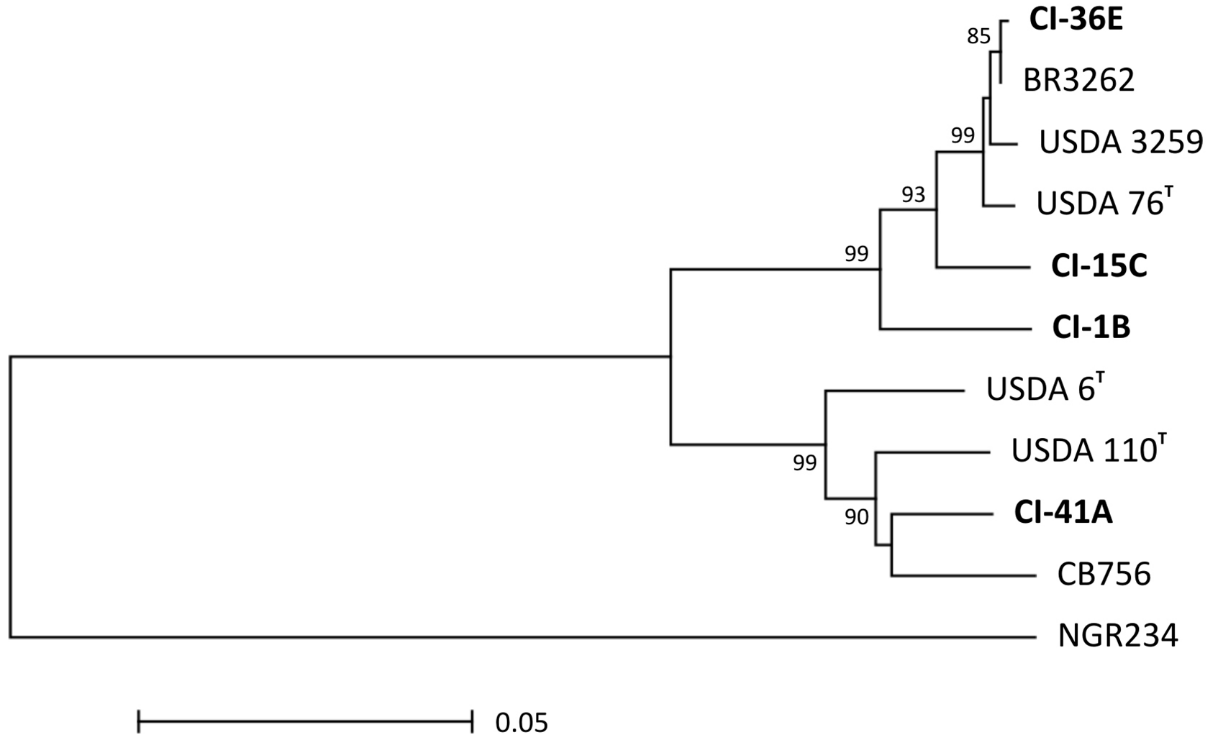

Legend to Figure S1. Phylogenetic tree was constructed using neighbour-joining method integrating the Tamura-Nei + Gamma (TN93+G) parameters with bootstrap values issued from 1,000 repetitions and shown only for those ≥70. Note that as in the 16S rRNA-ITS-23S rRNA tree, the reference rhizobial strains *B. elkanii* USDA 76^{T}, USDA 3259 and *B. pachyrhizi* BR3262 are found between CI-36E and CI-15C, both of which are cluster I nodule isolates.

Figure S2. Symbiotic phenotypes of CI-1B, -36E, CI-41A and NGR234 on *C. cajan* cv. ILRI 16555 grown in Magenta jars.

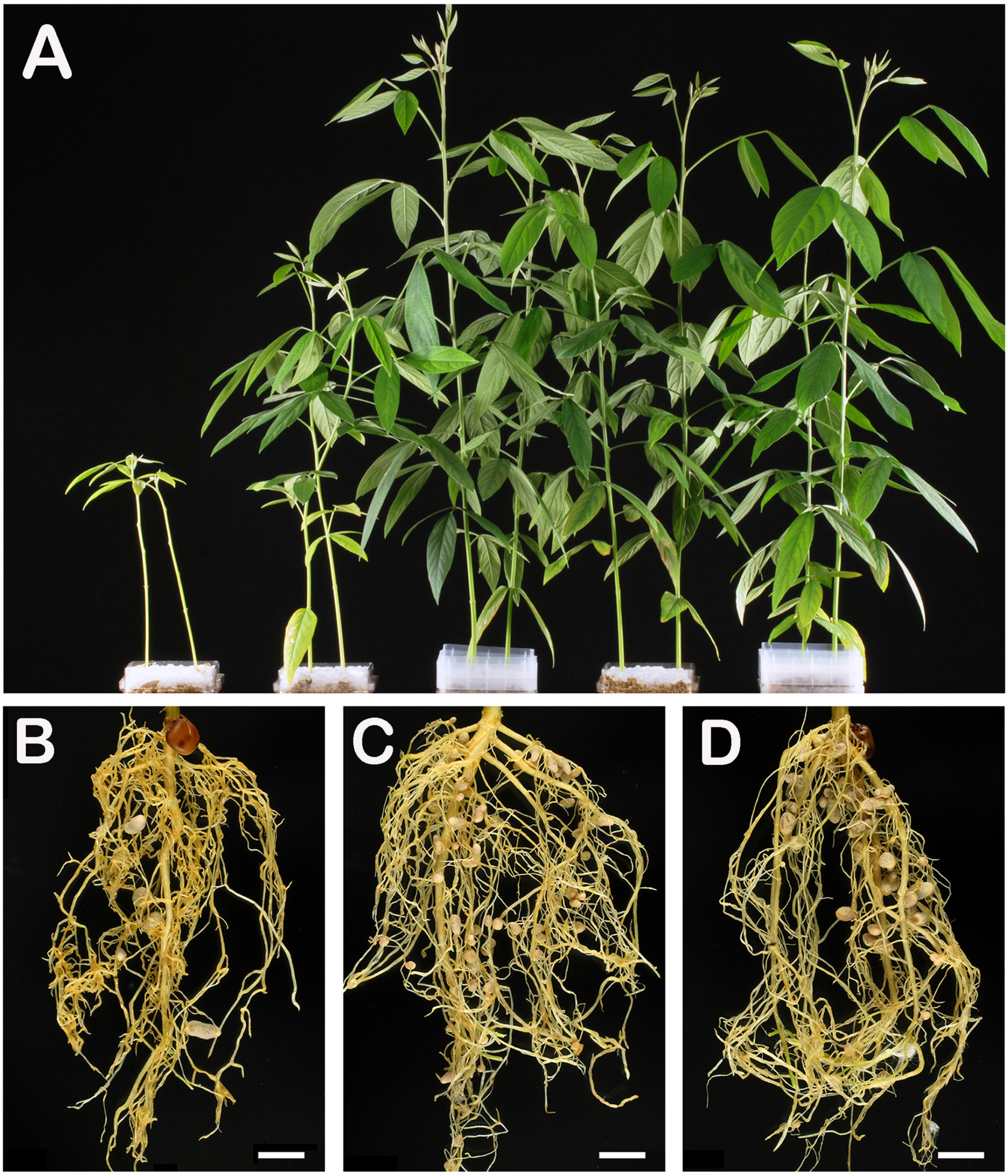

Legend to Figure S2. Panel **A**, shoots of *C. cajan* cv. ILRI 16555 plants 42 dpi with, from left to right, no inoculum, NGR234, CI-1B, -36E and CI-41A. Details of root systems of those same plants inoculated with either NGR234 (panel **B**), CI-1B (**C**) and CI-41A (**D**).

Figure S3. Scanning electron micrographs of whole cells of isolates CI-1B, CI-5B, CI-36E and CI-41A.

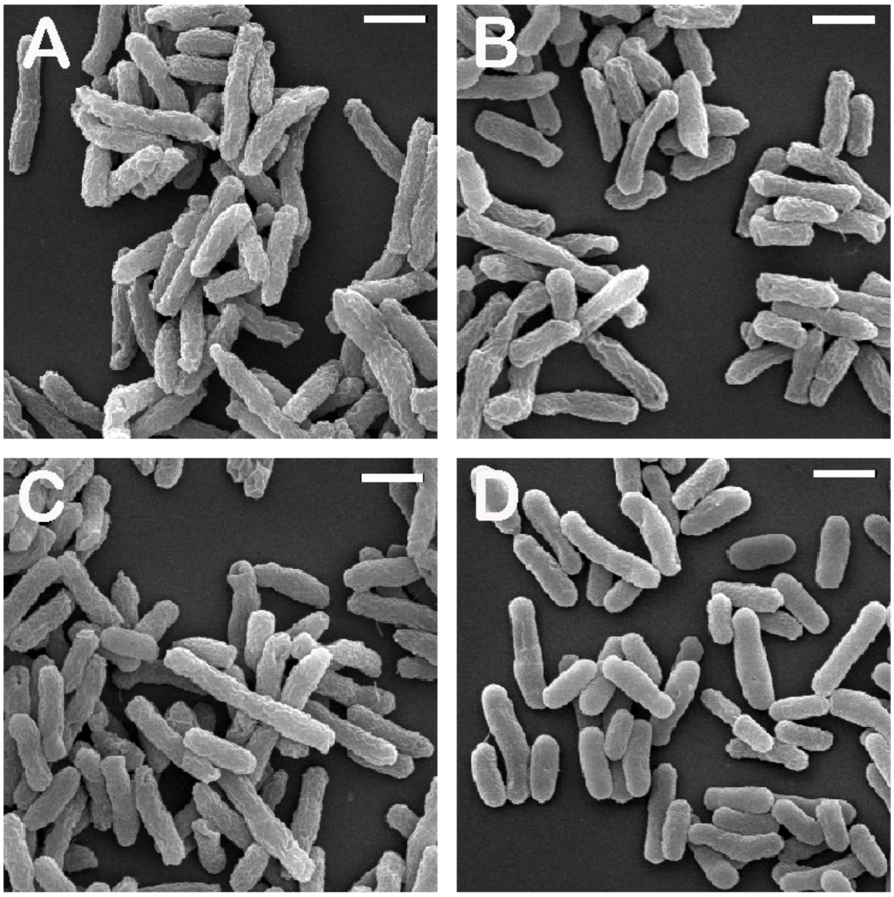

Legend to Figure S3. Cells of the symbiotic isolates CI-1B (A), CI-36E (B) and CI-41A (C) as well as the non-symbiotic CI-5B (D) are shown at the same magnification with a scale bar of 1 μm.

Table S1. Mass spectral identification, field of origin and known characteristics of nodule isolates.

	MALDI-TOF MS ident.	Field	Isolate	Growth	Best match to ITS
1	*Bradyrhizobium elkanii* (Cluster I)	1	**CI-1A**	S	*Bradyrhizobium pachyrhizi* strain BR3262
2			CI-4A1	S	
3			CI-5D	S	
4			CI-5E	S	
5		2	**CI-7A**	S	*B. pachyrhizi* strain BR3262
6			CI-7B	S	
7			CI-7C	S	
8			CI-7E	S	
9			CI-8A	S	
10			CI-8E	S	
11			CI-10B	S	
12			CI-10C	S	
13		3	**CI-15C**	S	*Bradyrhizobium valentinum* strain LmjM6
14		4	CI-18D	S	
15			CI-19C	S	
16			**CI-19F**	S	*B. valentinum* strain LmjM6
17		5	CI-31C	S	
18			CI-31D	S	
19			**CI-33M**	S	*B. pachyrhizi* strain BR3262
20			CI-34B	S	
21			CI-35A1	S	
22			CI-35C	S	

23			CI-35D	S	
24			**CI-36B**	S	*B. pachyrhizi* strain BR3262
25			CI-36C	S	
26			**CI-36E**	S	*B. pachyrhizi* strain BR3262
27		6	CI-37C	S	
28			CI-38A	S	
29			CI-38B	S	
30			CI-38C	S	
31			CI-39A	S	
32			CI-39B	S	
33			CI-39C	S	
34			CI-39D	S	
35			CI-40A	S	
36			CI-40D	S	
37			CI-40E	S	
38			**CI-40F**	S	*B. pachyrhizi* strain BR3262
39			CI-41E2	S	
40			CI-41F	S	
41			CI-41G	S	
42			CI-41H	S	
43			CI-41K	S	
44	*Bradyrhizobium* sp. (Cluster II)	1	**CI-1B**	S	*B. elkanii* strain UASWS1015
45			CI-4A2	S	
46			CI-4A3	S	
47			CI-4C	S	
48			CI-4D	S	

49		3	**CI-14A**	S	*B. elkanii* strain UASWS1015
50			CI-14B	S	
51			CI-15A	S	
52			CI-15D	S	
53		4	CI-18C	S	
54			CI-19A1	S	
55			**CI-19D**	S	*B. elkanii* strain UASWS1015
56			CI-19E	S	
57		5	**CI-33F**	S	*B. elkanii* strain UASWS1015
58			CI-33K	S	
59			CI-35B	S	
60		6	CI-41B	S	
61			CI-41L	S	
62			**CI-41S**	XS	*B. elkanii* strain UASWS1015
63	*Enterobacter* sp.	1	CI-4B	M	
64	*Sphingomonas xenophagum*		**CI-5B**	M	*Rhizobium alamii* strain YR584
65	not identified	4	**CI-16A**	M	*Paenibacillus* sp. strain JDR-2
66	not identified	5	CI-32E1	S	
67	not identified		CI-33L	S	
68	not identified		**CI-34D1**	M	*Brevibacillus reuszeri* strain DSM 9887
69	not identified		**CI-34F**	S	*Bacillus soli* strain NBRC 102451
70	*Staphylococcus lugdunensis*	6	CI-37E2	M	
71	*Rhizobium gallicum*		**CI-39Bx**	S	*Rhizobium* sp. strain JGI 0001005-K05
72	*Bradyrhizobium japonicum*		**CI-41A**	XS	*Bradyrhizobium* genosp. strain CB756
73	*Micrococcus luteus*		CI-41E1	S	
74	not identified		CI-41J1a	M	

75	*Bacillus megaterium*		CI-41J1b	M	
76	*Bacillus megaterium*		CI-41J2a	M	
77	not identified		CI-41J3	M	
78	not identified		CI-41M2	M	
79	*Rhizobium radiobacter*		CI-41P1	M	
80	not identified		CI-41P2	XS	
81	*Bacillus megaterium*		CI-41Q1	M	
82	not identified		CI-41Q3	S	
83	*Bacilus subtilis*		CI-41U2	M	
84	*Rhizobium radiobacter*		CI-41U3	M	
85	*Pseudomonas putida*		CI-41U4	M	

Legend to Table S1. The 85 strains isolated from pigeon pea nodules were listed according to the identification and clustering obtained with mass spectrometry and the field in which the source nodule was collected. Isolate numbering includes plant (numeral) and nodule of origin (letter): For example, the four isolates CI-7A to CI-7E were all collected on roots of plant 7 but from nodules A, B, C or E. Growth time on RMS and at 27°C required for obtaining ca. 1 mm diameter single colonies during isolate purification was reported as fast (F, less than 2 days incubation), medium (M, 2 to 5 days), slow (S, 6 to 14 days) or extra slow (XS, >14 days). For isolates that were further characterized by DNA sequencing (shown in bold), corresponding ITS sequences were matched against fully sequenced bacterial genomes archived in GenBank non-redundant or whole-genome shotgun contigs (in April 2016), with only the best match reported. For isolates CI-16A, -34D1, CI-34F only 16S rDNA sequences were used for BlastN analyses.

Table S2. List of primers used and sequences obtained during this study.

**Primer**	**5' to 3' sequence**	**Size** (bases)	**Feature**	**Reference**
16S-For3	agagttggatcctggctcag	20	*Bam*HI site	this work
16S-Rev3	aaaggaggggatccagccg	19	*Bam*HI site	this work
ITS-For2	tacacaccgcccgtcacacc	20	/	this work
ITS-Rev2	tggtccgcgttcgctcgcc	19	/	this work
NifH-For11	aagtcgaccacttcgcagaac	21	*Sal*I site	this work
NifH-Rev10	ccgtaatcgatcagcatgtcctc	23	*Cla*I site	this work
RpoB-For1	aacggatccgagcgcgtcatc	21	*Bam*HI site	this work
RpoB-Rev1	cgaggatccgaagaactcgcg	21	*Bam*HI site	this work

**Sequence**	**Strain**	**Size** (bp)	**ITS** (bp)	**Accession number**	**Reference**
16S-ITS-23S	CI-1A	2479	801	KX396569	this work
16S-ITS-23S	CI-1B	2540	862	KX396570	this work
16S-ITS-23S	CI-5B	2764	1054	KX396571	this work
16S-ITS-23S	CI-7A	2479	801	KX396572	this work
16S-ITS-23S	CI-14A	2540	862	KX396573	this work
16S-ITS-23S	CI-15C	2588	910	KX396574	this work
16S-ITS-23S	CI-19D	2539	861	KX396575	this work
16S-ITS-23S	CI-19F	2588	910	KX396576	this work
16S-ITS-23S	CI-33F	2540	862	KX396577	this work
16S-ITS-23S	CI-33M	2479	801	KX396578	this work
16S-ITS-23S	CI-36B	2480	802	KX396579	this work
16S-ITS-23S	CI-36E	2479	801	KX396580	this work
16S-ITS-23S	CI-39Bx	2589	953	KX396581	this work
16S-ITS-23S	CI-40F	2479	801	KX396582	this work
16S-ITS-23S	CI-41A	2467	760	KX396583	this work
16S-ITS-23S	CI-41S	2540	862	KX396584	this work

**Sequence**	**Strain**	**Size** (bp)	**Accession number**	**Reference**
16S	CI-16A	1431	KX396552	this work
16S	CI-34D1	1424	KX396553	this work
16S	CI-34F	1406	KX396554	this work
nifH	CI-1A	729	KX396555	this work
nifH	CI-1B	729	KX396556	this work
nifH	CI-7A	729	KX396557	this work
nifH	CI-14A	729	KX396558	this work
nifH	CI-15C	729	KX396559	this work
nifH	CI-19D	729	KX396560	this work
nifH	CI-19F	729	KX396561	this work
nifH	CI-33F	729	KX396562	this work
nifH	CI-33M	729	KX396563	this work
nifH	CI-36B	729	KX396564	this work
nifH	CI-36E	729	KX396565	this work
nifH	CI-40F	729	KX396566	this work
nifH	CI-41A	729	KX396567	this work
nifH	CI-41S	729	KX396568	this work
rpoB	CI-1B	923	KX388393	this work
rpoB	CI-15C	923	KX388394	this work
rpoB	CI-36E	923	KX388395	this work
rpoB	CI-41A	923	KX388396	this work

Legend to Table S2. Primers 16S-For3 and 16S-Rev3 cover respectively positions 10 to 27 and 1524 to 1542 of the *Escherichia coli* 16S rRNA. ITS-For2 and ITS-Rev2 anchored respectively into 3'-end of the 16S rRNA (pos. 1393 to 1412 of *E. coli* 16S rRNA) and 5'-end of 23S rRNA (pos. 265 to 247 of *E. coli* 23S rRNA) genes.

Table S3-A. Summary of symbiotic properties of selected nodule isolates on tested legume hosts. Phenotypes are reported as the ability of a strain to induce nodule formation on (Nod+) and fix nitrogen with (Fix+) a given host. NT, not tested. *Only pseudonodules formed. Unlike for pigeonpea and siratro (42 dpi), *T. vogelii*, cowpea and *L. leucocephala* plants were harvested at 45, 30 and 49 dpi, respectively.

	Cajanus cajan cv.		*M. atropurpureum* cv. Siratro	*T. vogelii*	*V. unguiculata* cv. Red Caloona	*L. leucocephala*
Inoculum	ILRI 16555	“Light Brown”				
CI-1B	Nod+ / Fix+	Nod+ / Fix+	Nod+ / Fix+	Nod+ / Fix+	Nod+ / Fix+	Nod+* / Fix-
CI-5B	Nod- / Fix-	Nod- / Fix-	NT	Nod- / Fix-	Nod- / Fix-	NT
CI-36E	Nod+ / Fix+	Nod+ / Fix+	Nod+ / Fix+	Nod+ / Fix+	Nod+ / Fix+	Nod+*/ Fix-
CI-39Bx	Nod- / Fix-	Nod- / Fix-	NT	NT	NT	NT
CI-41A	Nod+ / Fix+	Nod+ / Fix+	Nod+ / Fix+	Nod+ / Fix+	Nod+ / Fix+	Nod+* / Fix-

Table S3-B. Phenotypes of selected strains on *Vigna radiata* cultivar King after 35 days of growth in controlled conditions and reported as the mean nodule number (mNN), nodule fresh weight (mNFW) and shoot dry weight (mSDW) per plant. Pair of values found to be statistically different at the 5% level share the same superscript letter. Non-inoculated plants had mSDW of 210.0 (±9.9) mg.

	V. radiata cv. King			
Inoculum	Plants	mNN	mNFW (mg)	mSDW (mg)
CI-1B	4	168.0 (±30.8)^{a,b}	1463.0 (±342.8)^{f}	2312.0 (±638.5)^{i}
CI-15C	4	259.0 (±38.1)^{a,c,d}	1653.0 (±154.7)^{g}	3188.3 (±474.3)^{j}
CI-36E	4	2.5 (±2.7)^{b,c,e}	77.5 (±112.8)^{f,g,h}	230.3 (±115.4)^{i,j,k}
CI-41A	4	179.0 (±21.9)^{d,e}	1440.5 (±292.4)^{h}	3070.0 (±674.9)^{k}

Table S4. Major physical and chemical properties of the 2 to 20 cm deep soil fractions collected in fields #1, 2 and 4.

	Field n°		
Soil texture	1	2	4
USDA Class	Sandy clay loam	Sandy loam	Sandy loam
% Clay	28.8	13.6	15.8
% Sand	46.6	69.4	76.1
% Silt	24.6	17.0	8.1
Soluble elements (mg/kg)			
Ca	25.0 p	35.0 s	10.0 p
Cu	0.2	0.2	0.2
Fe	42.9	32.2	16.7
K	19.7 r	87.1 r	31.5 s
Mg	7.6 m	10.3 s	3.0 p
Mn	0.6	0.6	0.2
P	1.5 m	4.6 m	1.6 p-m
Zn	0.2	0.3	0.2
Other parameters			
Soil pH (H_2O)	6.4	7.2	6.3
Organic matter (%)	3.1 s	2.5 r	1.7 m-s
Total nitrogen (%)	0.2	0.1	0.1
Carbon/nitrogen ration	10.0	12.1	9.9
Available nitrogen (kg/ha)	20.4	45.7	21.3

Legend to Table S4. Physical and chemical properties were measured by Soil-Conseil (Gland, Switzerland) using standard methods (ISO 17025). Overall soil texture was based on the USDA particle-size classification. Depending on texture and major characteristics of each soil, a number of values (Ca, K, Mg, and P concentrations) were assessed as poor (p), mediocre (m), adequate (s) or rich (r).
